# The mercurial rise in research of halide perovskites: what´s next

**DOI:** 10.1007/s42247-024-00834-7

**Published:** 2024-10-15

**Authors:** Mohd Taukeer Khan, Shahzada Ahmad

**Affiliations:** 1 Department of Physics, Faculty of Science, Islamic University of Madinah, Prince Naifbin Abdulaziz Road, Al Jamiah, Madinah, 42351 Kingdom of Saudi Arabia; 2 BCMaterials, Basque Center for Materials, Applications, and Nanostructures, UPV/EHU, Science Park, Leioa, 48940 Spain; 3 IKERBASQUE, Basque Foundation for Science, Bilbao, 48009 Spain

## Abstract

Perovskites are of high potential in the ongoing academic research, due to their distinctive electrical properties and crystalline structures. Halide perovskites show high light emissive properties and panchromatic light absorption across the visible spectrum. The exceptional electrical characteristics, such as their long carrier lifespan, high diffusion length, and charge carrier mobility, allow the electric charges to be transported and collected effectively. Furthermore, by tuning the cations and anions composition, perovskite’s opto-electrical properties can be altered. Moreover, dimension reduction affects their band gap and intrinsic features to induce higher structural stability but at the cost of the quantum confinement effect. Owing to their exceptional properties, halide perovskites are being researched in energy-related and semiconducting applications, hold high promise and the future looks bright. But challenges remain, and the larger question is what needs to be done to make them more stable.

Ongoing academic research recognizes perovskites as a subject of considerable potential due to their intricate and unique crystalline structures and electrical merits. Metal halide perovskites (MHP) show the ability to absorb panchromatic light across the visible spectrum and also emit light with high efficacy. They possess remarkable electrical properties, including high charge carrier mobility, extended diffusion length, and long carrier lifetime, which induce the transportation of electric charges over considerable distances [1]. Moreover, the opto-electrical characteristics of MHP can be modulated through the compositional engineering of their constituent. The structural dimensionality of perovskites significantly influences their band gap and other inherent properties, with reduced dimensionality. Owing to remarkable optical, electrical, and photophysical properties, MHP finds their application in various energy-related and semiconducting applications (Fig. 1) [2]. Perovskite thin films have significant value in optoelectronics, laser technology [3], artificial synaptic [4], and pressure-sensitive systems [2]. They also play a role in catalysis [5], energy conversion in fuel cells [6], a range of sensing technologies [7], data storage [8], and ultrasound imaging [9]. These materials create light-emitting substances under stress, opening avenues for innovative sensors and displays. Serving as catalysts, perovskites can reduce energy consumption and increase chemical process efficiency. Through the photochemical approach, perovskites assist in reducing emissions or waste pollutants and transforming solar energy into chemical energy. They are being utilized to develop gas and chemical sensors, contributing to environmental surveillance. In electronics devices, they show promise for non-volatile memory devices crucial for maintaining data without power. Additionally, perovskites are key in spintronics to develop effective computing and are investigated for their acoustic properties in ultrasonic and underwater uses.

**Fig. 1:**
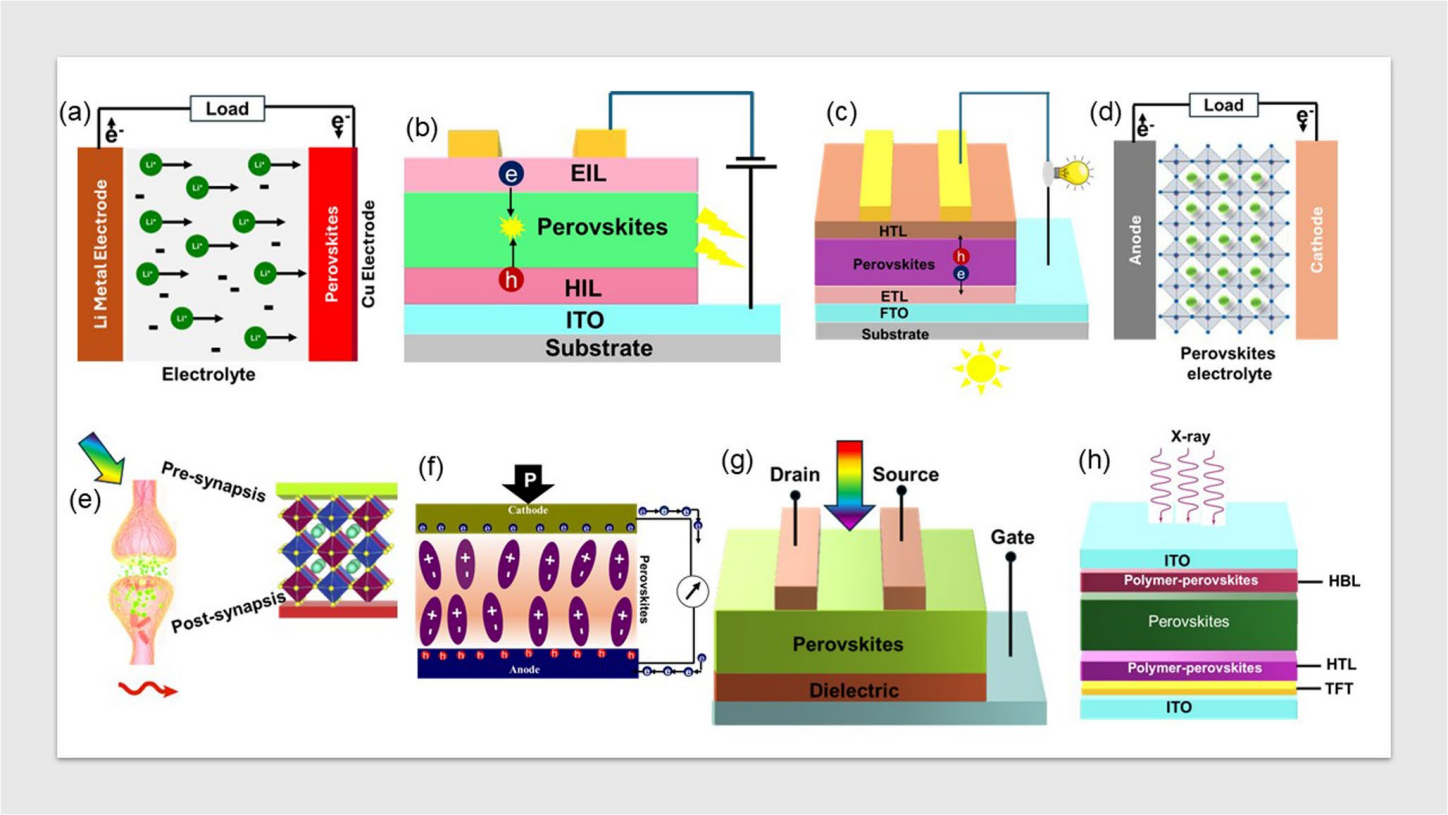
Illustrations of devices incorporating halide perovskites: (**a**) battery, (**b**) light-emitting diode, (**c**) solar cell, (**d**) supercapacitor, (**e**) artificial synaptic [4], (**f**) piezoelectric sensor, (**g**) photodetector, and (**h**) X-ray detector. (**e**) reproduced from Ref. 4 of Wiley (Creative Commons Attribution License)

In photovoltaics, perovskite has emerged as a formidable contender to conventional silicon-based solar cell technology in terms of performance. Over the past decade, concerted research and development efforts have culminated in a significant enhancement of power conversion efficiencies, now surpassing 26% [10]. This remarkable advancement is primarily attributed to the intrinsic properties of perovskites, such as panchromatic light absorption capabilities, and electrical merits. Additionally, these improvements are attributed to upgraded perovskite thin-film quality achieved through novel synthesis routes and optimized interface for device fabrication using innovative charge selective layers. The adaptability of perovskite is further evidenced by their successful incorporation into a multitude of device architectures. These include but are not limited to, planer n-i-p (n-type/intrinsic/p-type), inverted p-i-n (p-type/intrinsic/n-type), and mesoscopic configurations. The scope of perovskite application extends to an array of tandem solar cell designs, incorporating silicon-perovskite, perovskite-perovskite, CIGS-perovskite, and organic-perovskite combinations, manifested in two-terminal (2T), three-terminal (3T), and four-terminal (4T) configurations [11]. These advancements attest to the potential of perovskite solar cells (PSCs) to play a pivotal role in the future landscape of renewable energy sources.

The emergence of perovskite-based photodetectors represents a transformative advancement in the field, distinguished by their unparalleled sensitivity, swift response rates, enhanced stability, and comprehensive detection spectrum. The strategic incorporation of low-dimensional perovskite architectures, including two-dimensional (2D), one-dimensional (1D), and zero-dimensional (0D) configurations, has been pivotal in reducing recombination rates and improving charge transport. These enhancements significantly increase the sensitivity and accelerate the response times [12]. Further improvements in sensitivity and detectivity have been achieved through interface engineering and surface passivation, which can reduce trap states and in turn improve charge carrier dynamics, leading to an increase in performance metrics [13]. The development of multifunctional perovskite based photodetectors, which can detect polarized light, leverages the anisotropic properties of perovskite to achieve heightened sensitivity to the polarization state of incident light [14]. Moreover, the engineering of perovskite photodetectors with tunable spectral response enables coverage across an extensive wavelength range, which is crucial for spectroscopy and environmental monitoring applications. The advent of self-powered perovskite photodetectors eliminates the dependence on external power sources by utilizing the photovoltaic effect, making them suited for remote sensing applications. These perovskite-based photodetectors delivered effective metrics with high sensitivity, surpassing traditional semiconductor photodetectors, with responsivity values reported up to 748 A/W, significantly exceeding the 0.6–0.8 A/W range typical of silicon photodetectors in the visible spectrum [15, 16]. The external quantum efficiencies (EQE) values have risen to 254%, a stark contrast to the 80 − 90% typically achieved by silicon counterparts. Moreover, specific detectivity values have peaked at 8.2 × 10^12^ Jones, shadowing the 10^11^ to 10^12^ Jones for silicon photodetectors. Noise equivalent power values are generally lower for perovskite based photodetectors, indicating higher sensitivity, in contrast to silicon based photodetectors with higher values [16, 17]. Response times for perovskite photodetectors are in the range of nanoseconds, suitable for high-speed applications, compared to microseconds for silicon photodetectors [12, 16]. Such attributes make perovskite photodetectors promising for applications in imaging, optical communication, and environmental monitoring [18, 19].

MHPs have also garnered attention as potential materials for memristor devices in neuromorphic computing systems, demonstrating proficiency in pattern recognition, adaptive learning, and real-time data processing [20–22]. MHP possesses characteristics, including rapid ion migration for swift switching, adjustable composition for optimized performance, high defect tolerance for reliable operation, and exceptional optoelectronic properties to facilitate the integration of optical and electronic functionalities. Artificial synapses, which are indispensable components of neuromorphic systems, emulate the synaptic plasticity and learning capabilities of the human brain. MHPs enable the development of synaptic devices that modulate conductance states in response to stimuli, promoting high-speed functionality with minimal energy expenditure [23]. Additionally, memristors employ ion migration and charge-trapping mechanisms to imitate synaptic activity, allowing for simultaneous information storage and processing. Recent reports introduced photon-mediated switching, memory, and neuromorphic operations, significantly improving device velocity and efficiency [24]. Structural and compositional tuning of MHPs have advanced scalable incorporation into neuromorphic architectures, augmenting both structural and optoelectronic attributes [25].

In the realm of light-emitting diodes (LEDs) and lasers, perovskite emerged as a choice of materials offering tunable bandgaps that facilitate a spectrum of colors, enhanced luminosity, and reduced energy demands for display technologies [26]. Preliminary reports on electroluminescence [27] and lasing action [28] from hybrid perovskites were reported in the 1990s. Over the past decade, MHP gave notable emitting properties, achieving EQE of 28.7% for LEDs and establishing low threshold values for laser operations [29, 30]. Such merits promise substantial improvements in brightness and spectral range, encompassing red, green, and blue emissions [31]. The introduction of polarization-controlled chiral emissions showed future promise in this direction [30]. Perovskite based LEDs (PLEDs) outshine their organic counterparts by a factor of a thousand, suggesting their suitability for perovskite injection lasers in diverse fields such as image projection, environmental monitoring, and medical diagnostics [32]. New developments continue to push the efficiency and durability of blue PLEDs [33], while 2D perovskites and their heterostructures present enhanced optical and electronic characteristics, along with boosted stability in ambient conditions [34]. The achievement of room-temperature continuous-wave lasing in perovskite films signifies rapid progress relative to other non-epitaxial semiconductors [35]. Moreover, the development of polarization-controlled chiral emissions broadens the scope of perovskites, potentially revolutionizing applications in spintronics and quantum computing.

Chiral perovskites are vouched as transformative materials for optoelectronic applications, owing to their distinctive ability to modulate electron spin via chirality-induced spin selectivity (CISS). The incorporation of chiral MHP with III-V optoelectronic semiconductors has culminated in the development of spin-LEDs, capable of emitting circularly polarized light at ambient conditions without external magnetic fields [36, 37]. Such a family of perovskites is promising in augmenting the efficiency and broadening the functionality of optoelectronic apparatuses, particularly by facilitating new operational modes in LEDs and photovoltaic cells (NREL News). The dual-ligand quasi-layered perovskites notably improved spin-LED performance at room temperature, propelling these devices closer to widespread practical deployment [38]. Furthermore, chiral perovskites are poised to revolutionize X-ray imaging technology. Their scintillators emit circularly polarized radioluminescence, enabling the production of X-ray imagery with diminished optical crosstalk, thereby enhancing image clarity and resolution [39]. Perovskite-based X-ray detectors delivered high sensitivity and spatial resolution, rendering them suitable for medical diagnostics and other precision-dependent X-ray detection applications. The tuning of radioluminescence propagation pathways in chiral perovskites represents a significant step in the enhancement of X-ray imaging device performance [40]. The employment of chiral perovskites in optoelectronic devices, alongside their burgeoning role in X-ray imaging, highlights their pivotal role in the advancement of materials science.

Despite significant advancements in performance, challenges hinder the widespread adoption of perovskite-based devices, including stability under environmental conditions, toxicity, scalability, and maintaining efficiency at larger scales [41]. Among these, stability is of main concern [42], especially in bulk (3D) perovskites. The stability of perovskites depends on both intrinsic and extrinsic factors (Fig. 2). Key components contributing to intrinsic stability include the perovskite active layer, recombination layers, charge transport layers (CTL), and contact electrodes [6]. These materials are sensitive to extrinsic factors such as heat, light, and humidity, which can accelerate degradation through various interface formations or by-product formation due to chemical interactions. The compatibility between perovskite and CTL, as well as contact electrodes, significantly influences device reliability. One critical factor is the presence of volatile organic cations at the A sites within the perovskite lattice. These cations contribute to the material’s instability and susceptibility to degradation. Researchers have explored mixed cation perovskites (e.g., Cs_x_MA_y_FA_1−x−y_PbX_3_) to demonstrate enhanced thermal stability and superior device performance due to extended photoexcited species lifetimes and robust interactions between organic and inorganic components [43]. Additionally, PSCs exhibit anomalous behavior concerning voltage scan direction and speed. Variations in performance depending on the scan direction have been reported, stemming from unoptimized crystal formation remains an area of active investigation [44]. Moreover, perovskites undergo phase transitions under specific conditions. For instance, methylammonium lead triiodide exhibits phase transitions from the tetragonal to the orthorhombic phase. Similarly, formamidinium lead triiodide undergoes an unfavorable phase transition from a photoactive black-colored α-phase (*E*_g_ ~1.47 eV) to a photo-inactive yellow-colored δ-phase (*E*_g_ ~ 2.43 eV) at room temperature. These phase transitions impact its electronic band structure and subsequently deteriorate device performance. Another significant factor contributing to perovskite instability is the low activation energy for ion migration, particularly halide ions. Halide vacancies generated within the perovskite layers facilitate moisture penetration and ion diffusion, ultimately affecting device performance [45–47].

Challenges remain in achieving electrically driven laser diodes due to the high threshold carrier density required for lasing action [48]. Although direct evidence for electrically driven lasing is lacking, synchronized optical and electrical pumping in PLEDs has yielded threshold lasing current density estimates close to current achievements. Furthermore, the low thermal conductivity of perovskites poses a hurdle for continuous operation, as efficient heat dissipation is crucial for maintaining performance and extending the laser’s lifespan. Non-radiative losses at high-charge carrier injections can diminish laser efficiency. The stability of perovskite lasers is often compromised by high threshold carrier densities and thermal management issues. Furthermore, interactions with metals like gold and silver can negatively affect the material’s properties.

**Fig. 2:**
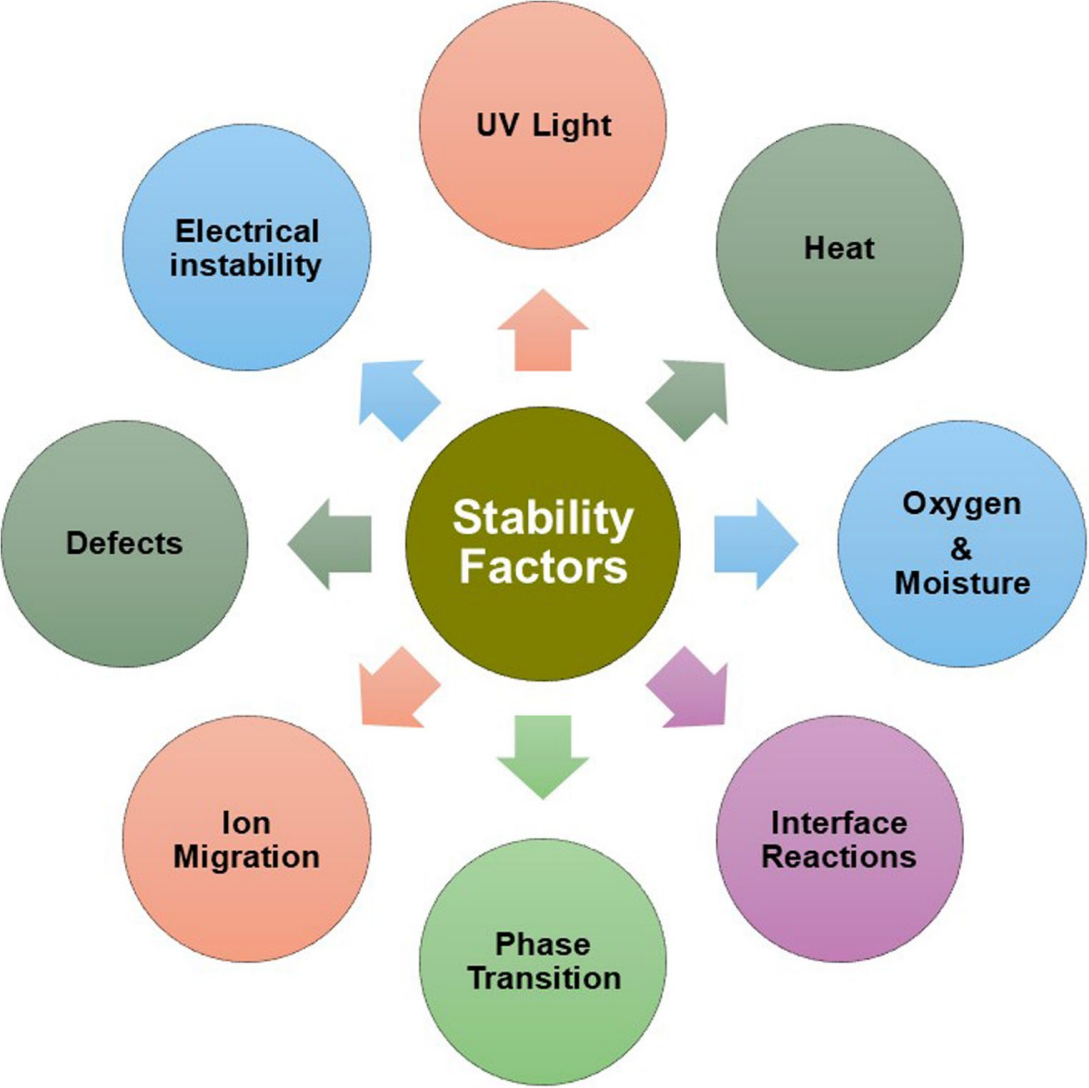
Factor influencing the stability of perovskites-based devices

To stabilize perovskite-based devices, both intrinsic and extrinsic approaches are actively pursued. Robust encapsulation techniques are paramount for protecting perovskite from external factors such as heat, moisture, and oxygen. The application of flexible surface layers possessing hydrophobic characteristics, such as the block copolymer F127, shield perovskite layers from moisture and oxygen intrusion, extending their operational lifetime [49]. Hybrid (organic and inorganic materials) encapsulation layers, provide robust protection against environmental degradation, enhancing stability under various stress conditions [50]. Molecular modulators or passivators encapsulate grain boundaries to prevent moisture and oxygen ingress, maintaining the integrity of the perovskite structure over time [51]. Improving intrinsic stability entails innovative techniques: structural and compositional engineering, choice of solvent, additives for grain growth control, interfacial passivation, the selection of stable charge transport materials, the development of layered and bulk perovskite layer structures, substituting organic components with inorganic counterparts, and tuning of device architectures. Surface passivation of perovskite reduces surface defects and minimizes non-radiative recombination. Large ammonium cations to passivate defects that reduces recombination also has been reported to improve, both efficiency and stability in PSCs [52]. These cations can form a thin molecular layer or a low-dimensional perovskite as a capping layer. 4-aminophenyl sulfone (APS) has been also introduced as a surface passivation agent, interacting with uncoordinated Pb^2+^ ions, reducing trap density, and inducing a more p-type surface, resulting in higher power conversion efficiency and improved environmental and thermal stability [53]. Cyanomethyltriphenylphosphonium chloride (CTPC) molecules passivate undercoordinated lead sites by bonding the C = N group with Pb^2+^, thereby reducing interfacial defects and allowing improved performance and stability of planar PSCs [54]. Additive engineering, involves small amounts of materials incorporated into the perovskite structure, to improve stability. Additives such as cesium and rubidium enhance thermal and moisture stability by stabilizing the crystal structure and reducing defect formation [55]. Polymers and fullerene derivatives improve the morphology of perovskite films, leading to better crystallization kinetics and device performance by controlling grain boundaries and reducing defect densities [36]. Metal halide salts also push stability and efficiency by passivating defects by improving the overall quality of the perovskite layer [56]. Advances in device architectures can also impact the stability of perovskite-based devices. Empirical evidence frequently suggests superior performance metrics associated with *n-i-p* configurations in solar cells, while higher stability is reported by *p-i-n* structures, this is mainly due to comprising stability of n and p-type layers in *n-i-p* structure, while in *p-i-n*, this is limited by the stability of n-type layers, which is organic or inorganic. The lower thickness of charge selective layers used in *p-i-n* type solar cells, not only improves the stability but also minimizes the notorious hysteresis behavior, and in most cases, often eliminates the need for doping. PLEDs have demonstrated significant stability improvements through encapsulation, surface passivation, and the use of robust charge transport layers [57].

Developing lead-free alternatives is essential for environmental sustainability and health considerations [58, 59]. Efforts to replace lead with non-toxic metals such as tin (Sn), bismuth (Bi), germanium (Ge), and antimony (Sb) have been explored [60–62]. Tin emerges as a promising Pb alternative, with MASnI_3_ exhibiting a narrower band gap and higher absorption coefficient than MAPbI_3_, enhancing their potential for photovoltaic applications [63]. However, Sn-based perovskites face significant in stability issues due to the rapid oxidation of Sn^2+^ to Sn^+4^ [64, 65]. In this context Bismuth based perovskites are promising candidates for optoelectronic applications due to their favorable properties and lower toxicity compared to traditional lead-based perovskites [66]. Efforts to enhance the performance of tin perovskites are focused on mitigating oxidation, while bismuth perovskites are being optimized for stability and optoelectronic efficacy [67]. Germanium and antimony based perovskites are also under investigation in X-ray detection and imaging [68, 69]. The continuous development and appetite for emerging semiconductors in this field suggest the exploration of lead-free perovskites in various technological applications.

Recent advancements suggests advancement in the stability of MHP, further, addressing key challenges will pave the way for perovskite-based devices in various fields. Future research aims to develop stable perovskite compositions and protective encapsulation techniques to ensure reliable performance over extended periods. Efforts in perovskite-based devices should focus on several key areas to address existing challenges and enhance their practical applications. Improving optical feedback, coupling efficacy, and reducing optical losses are paramount. Addressing phase instabilities, ion migrations, phase separation, electric field-induced quenching, and lasing death phenomena is of critical interest. A deeper understanding of switching mechanisms, including ion migration and charge trapping, is vital for reliable operation [70]. Doping is a trade-off, however, advancements in doping methods, rational charge transport layers, and effective heat management strategies will accelerate progress toward achieving electrically excited lasing action.

The ability to be solution-processable in perovskites allows for large-area fabrication, and low capital cost investment for scalability from lab to fab. In this vein, advances in material engineering and device architecture are key for scaling up production while maintaining high uniformity in the deposit thin film. Though significant laboratory-scale achievements have been made, translating these into large-scale production requires overcoming challenges related to material uniformity, device integration, and thermal management. Ensuring uniformity and reproducibility of perovskite thin films necessitates further research into scalable, cost-effective fabrication processes compatible with existing semiconductor manufacturing technologies. Development of new protocols in addition to current scaling up roll-to-roll processing for high-throughput manufacturing, solution-based synthesis methods adaptable for mass production, and wet chemistry techniques like blade coating, slot-die coating, and inkjet printing. Vacuum deposition techniques like thermal evaporation may allow precise control of thin film thickness, promoting higher reproducibility, though their utility on a large scale is yet to be established. Automated manufacturing can enhance precision, reduce human error, and increase production speed, while material optimization focuses on developing stable, robust perovskite. Leveraging existing semiconductor manufacturing infrastructure can help scale up production more efficiently and rapidly, and ensuring environmentally friendly and safe production processes is paramount. Integration with existing semiconductor technologies and architectures is critical, involving compatibility with current fabrication processes and electronic systems. Addressing the environmental impact of production and disposal through eco-friendly synthesis and recycling will further increase the acceptance of these materials across the sectors. Overall, addressing the challenges of stability, toxicity, scalability, and efficiency is of paramount interest for the widespread adoption of perovskite technology. Future research will focus on exploring new functionalities and mitigating environmental impacts, to make halide perovskite-based devices viable alternatives for practical applications, that can potentially revolutionize fields such as quantum computers, artificial intelligence, and adaptive learning systems.
